# Dual observers based sliding mode control for QUAVs with unknown disturbances and time varying delays

**DOI:** 10.1038/s41598-025-88511-5

**Published:** 2025-02-14

**Authors:** Chuanfu Liang, Yuanchun Ding, Falu Weng, Weidong Chen, Jiawei Li

**Affiliations:** 1 School of Electrical Engineering and Automation, Jiangxi University of Science and Technology, Ganzhou, Jiangxi 341000 China; 2 Ganzhou Key Laboratory of Industrial Safety and Emergency Technology; Jiangxi Provincial Key Laboratory of Safe and Efficient Mining of Rare Metal Resource, Jiangxi University of Science and Technology, Ganzhou, Jiangxi 341000 China

**Keywords:** quadrotor unmanned aerial vehicle, time-varying delays, unknown disturbances, sliding mode control, Aerospace engineering, Electrical and electronic engineering

## Abstract

This paper presents a dual-observers-based nonsingular fast terminal sliding mode control scheme for quadrotor unmanned aerial vehicles (QUAVs) with unknown disturbances and time-varying delays. Firstly, to facilitate the controller design, the QUAVs model is decoupled into two subsystems: position subsystem and attitude subsystem. Secondly, for the position subsystem, a sliding mode controller is presented to control the position of the QUAVs. For the attitude subsystem, by introducing an exponential term, a nonsingular fast terminal sliding mode controller is obtained to ensure the fast convergence of the attitude angles. Moreover, based on the exponential term, the singularity problem of the conventional terminal sliding mode is solved. Thirdly, the disturbance and time-varying delay observers are presented by considering the time-varying delayed signals and unknown disturbances. Finally, the effectiveness and feasibility of the proposed control scheme are demonstrated by some computer simulations.

## Introduction

In recent years, quadrotor unmanned aerial vehicles (QUAVs) were widely used in the aerial photography, wildfire monitoring, agricultural irrigation, and military missions, owing to their advantages, such as, vertical take-off and landing, hovering flight, etc^1–4^. It is well known that the QUAVs have the characteristics of highly nonlinear, strong coupling, and under-actuation. Furthermore, complex and changing environment during the process of the flight can also result in lots of unknown disturbances and uncertain factors to the QUAVs. These factors may not only cause the flight trajectory of the QUAVs to deviate from the predetermined path but may also trigger the instability of the flight attitude and even cause flight accidents. Fortunately, in the past several decades, some efforts were tried by scholars, and lots of results were achieved. For example, sliding mode control (SMC)^5^, backstepping control^6^, adaptive control^7^, etc., were all extended to the control of QUAVs, and their effectiveness were proofed by some numerical examples. In these works, the hierarchical inner-outer loop control strategy is a common approach to address the complex dynamics of QUAVs. Specifically, the control system of the QUAV is divided into an inner-loop attitude control system and an outer-loop position control system. To ensure the feasibility of the inner-outer loop control, an essential requirement is that the convergence rate of the inner-loop is faster than the outer-loop^8^. Among these achieved control schemes, the SMC was considered as an effective quadrotor control technique, which have a high degree of robustness in dealing with system uncertainties and external disturbances, and some SMC-based control methods were also achieved by the researchers in many areas. Such as, Shi et al.^9^ studied an integral SMC to attenuate the effects of the model uncertainty and external disturbances. Islam et al.^10^ proposed a nonlinear robust adaptive SMC algorithm to deal with the tracking control problem of a QUAV with some bounded uncertainty conditions. Weng et al.^11^ proposed a cascade controller that combines adaptive SMC with recursive techniques to control QUAVs with time-varying loads and unknown disturbances. However, the conventional SMC or SMC-based control methods can only guarantee asymptotic convergence of the systems and are still suffering from the chattering problem. In order to solve this problem, Wang et al.^12^ achieved a terminal sliding mode control (TSMC) for the trajectory tracking control of a QUAV, which allowed the tracking errors to converge to zero in finite time. Nevertheless, TSMC still faces two problems: one is that TSMC converges slower than SMC when the system state is far from the equilibrium point, and the other is that there may be a singularity problem existing in TSMC. Fortunately, Gao et al.^13^ developed a fast terminal sliding mode control (FTSMC) scheme for the attitude system of the QUAVs, which can achieved a faster convergence rate than the conventional TSMC. In order to solve the singularity problem, Uzair et al.^14^ proposed a nonsingular terminal sliding mode control (NTSMC) for QUAVs, and some improved performances were achieved in the presence of parameter variations and unknown disturbances in the systems.

In practice systems, the disturbances are often complex and random, thus, it is difficult to utilize only the robustness of SMC to combat the system uncertainties and unknown disturbances. It is well known that SMC is robust for matched uncertainty, while it remains a challenge to design SMC schemes for systems with mismatched uncertainty^15^. In order to deal with those complex disturbances, many researchers introduced observer techniques to suppress the system uncertainties and unknown disturbances. Aiming at the unknown disturbances existing in the real working environment of QUAVs, Ma et al.^16^ designed an extended-state-observer-based sliding mode controller to estimate the system’s input-output state information and total disturbances in real time. Weng et al.^17^ proposed an adaptive SMC method combined with linear extended state observers for controlling QUAVs subjected to disturbances. In the reference^18^, an adaptive backstepping sliding mode controller, which combined with a disturbance observer, was proposed for the control of QUAVs with unknown disturbances, and some improved results were achieved. Wang et al.^19^ presented a disturbance-observer-based adaptive fault-tolerant control strategy for a QUAV subject to multiple actuator faults, parametric uncertainties, and external disturbances.

On the other hand, another unavoidable factor that affects the stability of QUAVs is the measurement delays^20^. The attitude and position of the QUAVs are measured by inertial measurement units (IMU) and global positioning systems (GPS), respectively, thus the measurement delays are unavoidable in practical applications due to the presence of sampling time, processing time, and data transmission time^21^. In general, the measurement delays of the IMU are so small that it can be ignored. However, the measurement delays of the GPS are significant due to the heavy computational load and long transmission distances^22^. Thus, the system analysis and controller design with measurement delays considered is necessary and meaningful. In recent years, many scholars have considered the measurement delays and some results were achieved. For example, Su et al.^20^ developed a nonlinear controller for vertical take-off and landing (VTOL) aircraft that suffer from measurement delays. The controller applied the Pade approximation technique to transform the original controlled system into a delay-free augmented dimension system, which achieved the VTOL aircraft asymptotic tracking of the desired trajectories. Kanishke et al.^23^ used an extended Kalman filter to fuse the time-lagged position measurements from GPS with the synchronized attitude and angular velocity measurements from IMU to achieve the correction of quadrotor position information. It is worth pointing out that the measurement delays considered by most of the existing results are constant. However, in practical systems, the measurement delays are time-varying, obviously. Thus, obtaining some results with time-varying measurement delays considered, is still necessary. Fortunately, some scholars have considered this problem. For example, He et al. proposed a state observer for a VTOL aircraft with time-varying measurement delays in reference^24^. Ashutosh et al.^25^ designed a fractional-order sliding mode controller based on the delayed output observer and analyzed the stability of the closed-loop system using Lyapunov-Razumikhin theorem.

However, to the best of the authors’ knowledge, as to the unknown disturbances and time-varying measurement delays for QUAVs, the existing achievements are relatively few, and obtaining some results in this field is still necessary and meaningful. Motivated by the this problem, this paper studies the position and attitude tracking control problems of the QUAVs with the unknown disturbances and time-varying delays. To facilitate the design of the controller, the dynamical model of the QUAVs is divided into two subsystems: attitude subsystem and position subsystem. Moreover, the attitude subsystem is subjected to some unknown disturbances, and position subsystem is affected by both some unknown disturbances and time-varying delays. A dual-observers (DO) based nonsingular fast terminal sliding mode control (NFTSMC) scheme is developed for the QUAVs with unknown disturbances and time-varying delays. The main contributions of this paper are summarized as follows:

- A DO-based sliding mode controller is designed to control QUAVs with unknown disturbances and time-varying delays to make sure that the controlled system can track the desired trajectory.
- A time-varying delay observer with the impact of some unknown disturbances considered, is proposed to estimate the time-varying delay signals in the position subsystem.
- To ensure the fast convergence of the attitude loop, a new NFTSMC scheme is proposed by introducing an exponential term. The control scheme can not only achieved fast convergence but also avoided the singularity problem.

The rest of the paper is organized as follows: The dynamic model of the QUAVs is presented in Section 2. In Section 3, the design of the DO-based sliding mode controller is presented. Some comparative simulation results are shown in Section 4. The conclusion is given in Section 5.

## Dynamic model of QUAVs

**Fig. 1:**
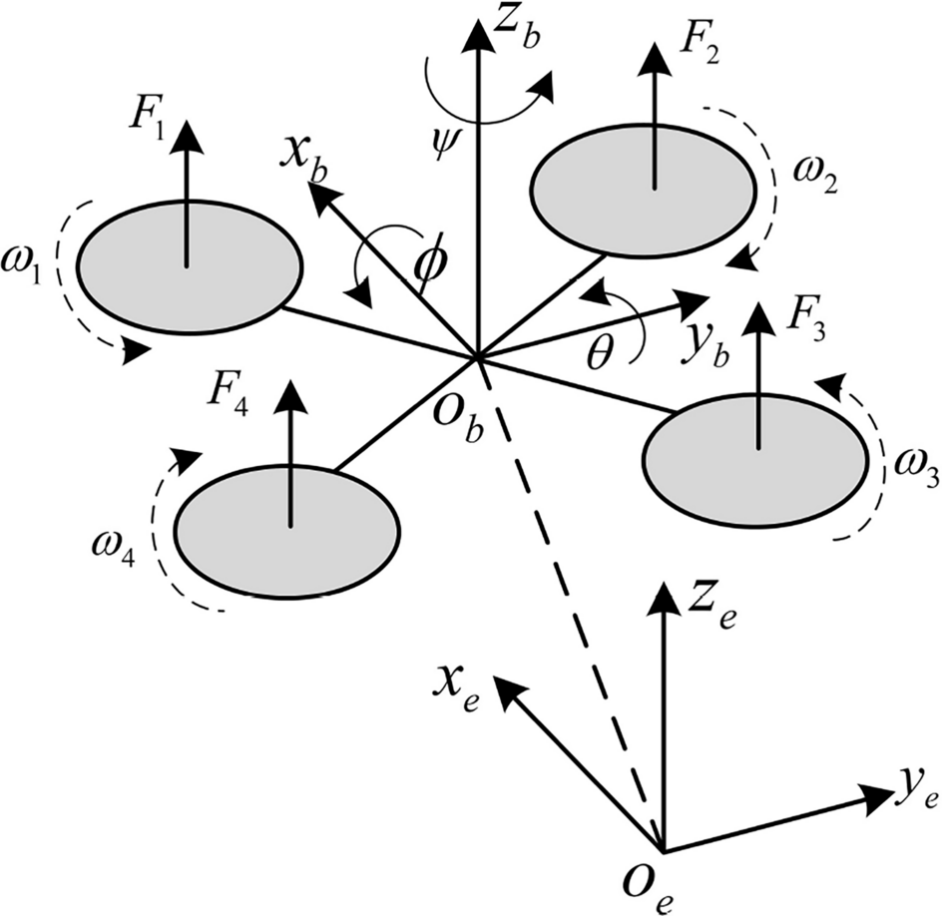
Structure of the QUAVs.

The structure of the QUAVs is illustrated in Fig. 1, and the attitude and position of the QUAVs can be changed by adjusting the angular rates of four propellers. In order to facilitate the system analysis of the QUAVs, the inertial coordinate system $\{ {o_e},{x_e},{y_e},{z_e}\}$ and the body-fixed coordinate system $\{ {o_b},{x_b},{y_b},{z_b}\}$ are introduced to describe the position $\{ x,y,z\}$ and attitude angles $\{ \phi ,\theta ,\psi \}$ of the QUAVs^26^. The Euler angles of the QUAVs are represented by the roll ($\phi$), pitch ($\theta$) and yaw ($\psi$), which are the rotating angles around the x-axis, y- axis, and z-axis, respectively. In order to simplify the mathematical model of the QUAVs, several assumptions are made as follows^27^:

### Assumption 1

The QUAVs are a rigid body model with an uniform mass distribution and a geometric center that coincides with the center of gravity.

### Assumption 2

The mass and rotational inertia of the QUAVs do not change with time.

According to the Newton’s second law and Euler’s dynamic equation, the dynamic model of the QUAVs are expressed as:^28^

1 $$\begin{aligned} & \left\{ \begin{aligned} \ddot{\phi }&= \frac{{{U_1}}}{{{I_1}}} - \frac{{{k_1}l}}{{{I_1}}}{\dot{\phi }} + {d_1}\\ \ddot{\theta }&= \frac{{{U_2}}}{{{I_2}}} - \frac{{{k_2}l}}{{{I_2}}}{\dot{\theta }} + {d_2}\\ \ddot{\psi }&= \frac{{{U_3}}}{{{I_3}}} - \frac{{{k_3}l}}{{{I_3}}}{\dot{\psi }} + {d_3}\\ \ddot{x}&= \frac{{{U_4}}}{m}(\cos \phi sin \theta \cos \psi + \sin \phi \sin \psi ) - \frac{{{k_4}\dot{x}}}{m} + {d_4}\\ \ddot{y}&= \frac{{{U_4}}}{m}(\cos \phi \sin \theta \sin \psi - \sin \phi \cos \psi ) - \frac{{{k_5}\dot{y}}}{m} + {d_5}\\ \ddot{z}&= \frac{{{U_4}}}{m}(\cos \phi \cos \theta ) - g - \frac{{{k_6}\dot{z}}}{m} + {d_6} \\ \end{aligned} \right. & \end{aligned}$$

where *l* is the distance from a propeller to the body of the QUAV; *m* is the total mass of the QUAV; *g* is the gravity acceleration; ${I_i}(i = 1,2,3)$ denote the moments of inertia; ${k_r}(r = 1, \cdots ,6)$ are drag coefficients; ${d_s}(s = 1, \cdots ,6)$ are various unknown external disturbances; ${U_1}$ , ${U_2}$ , ${U_3}$ and ${U_4}$ represent the control input signals in the roll, pitch, yaw and altitude channels, respectively, and satisfy the following equations:

2 $$\begin{aligned} \left\{ \begin{aligned} {U_1}&= \frac{{{k_T}l}}{{\sqrt{2} }}(\omega _1^2 - \omega _2^2 - \omega _3^2 + \omega _4^2)\\ {U_2}&= \frac{{{k_T}l}}{{\sqrt{2} }}(\omega _1^2 + \omega _2^2 - \omega _3^2 - \omega _4^2)\\ {U_3}&= {k_M}(\omega _1^2 - \omega _2^2 + \omega _3^2 - \omega _4^2)\\ {U_4}&= {k_T}(\omega _1^2 + \omega _2^2 + \omega _3^2 + \omega _4^2)\\ \end{aligned} \right. \end{aligned}$$

where ${k_T}$ is the thrust coefficient. ${k_M}$ is the torque coefficient. ${\omega _t}(t = 1, \cdots ,4)$ is the speed of the *i*th motor.

From the mathematical model of the QUAV, it can be seen that the horizontal position is an underactuated mechanical system. To control the underactuated part of the position subsystem, the virtual control inputs are selected as follows:

3 $$\begin{aligned} \left\{ \begin{aligned} {U_x}&= \frac{{{U_4}}}{m}(\cos \phi sin \theta \cos \psi + \sin \phi \sin \psi )\\ {U_y}&= \frac{{{U_4}}}{m}(\cos \phi \sin \theta \sin \psi - \sin \phi \cos \psi )\\ {U_z}&= \frac{{{U_4}}}{m}(\cos \phi \cos \theta ) \\ \end{aligned} \right. \end{aligned}$$

After a simple calculation, the altitude control input ${U_4}$, the desired pitch $\theta _d$ and roll $\phi _d$ angles can be obtained as:

4 $$\begin{aligned} \left\{ \begin{aligned} {\theta _d}&= \arctan \left(\frac{{{U_x}\cos {\psi _d} + {U_y}\sin {\psi _d}}}{{{U_z}}}\right)\\ {\phi _d}&= \arctan \left(\frac{{\cos {\theta _d}({U_x}\sin {\psi _d} - {U_y}\cos {\psi _d})}}{{{U_z}}}\right)\\ {U_4}&= \frac{{{U_z}m}}{{\cos {\theta _d}\cos {\phi _d}}}\\ \end{aligned} \right. \end{aligned}$$

To facilitate the design of the controller and avoid repetition, the dynamics model (1) of the QUAVs can be rewritten as:

5 $$\begin{aligned} \left\{ \begin{aligned}&{{\dot{x}}_{2i - 1}} = {x_{2i}}\\&{{\dot{x}}_{2i}} = f({X_{2i}}) + {b_i}{u_i} + {d_i}, \hspace{1em} i = 1,2,3\\ \end{aligned} \right. \end{aligned}$$

6 $$\begin{aligned} \left\{ \begin{aligned}&{{\dot{x}}_{2j - 1}} = {x_{2j}} \\&{{\dot{x}}_{2j}} = f({X_{2j}}) + {u_j} - {g_j} + {d_j} , \hspace{1em} j = 4,5,6\\ \end{aligned} \right. \end{aligned}$$

where ${[{x_1},{x_2}, \cdots ,{x_{12}}]^T} = {[\phi ,\dot{\phi },\theta ,{\dot{\theta }} ,\psi ,{\dot{\psi }} ,x,\dot{x},y,\dot{y},z,\dot{z}]^T}$ is the state vector; ${[f({X_2}),f({X_4}), \cdots f({X_{12}})]^T} = [ - {k_1}l{x_2}/{I_1}, - {k_2}l{x_4}/{I_2},$ $- {k_3}l{x_6}/{I_3}, - {k_4}{x_8}/m, - {k_5}{x_{10}}/m, - {k_6}{x_{12}}/m{]^T}$ are the nonlinear terms; ${[{b_1},{b_2},{b_3}]^T} = {[1/{I_1},1/{I_2},1/{I_3}]^T}$ are the weighting factors. ${[{u_1},{u_2}, \cdots ,{u_6}]^T} = {[{U_1},{U_2},{U_3},{U_x},{U_y},{U_z}]^T}$ are the control inputs of the attitude subsystem and position subsystem. ${[{g_4},{g_5},{g_6}]^T} = {[0,0,\mathrm{{g}}]^T}$ is the gravity acceleration vector.

## Controller design

**Fig. 2:**
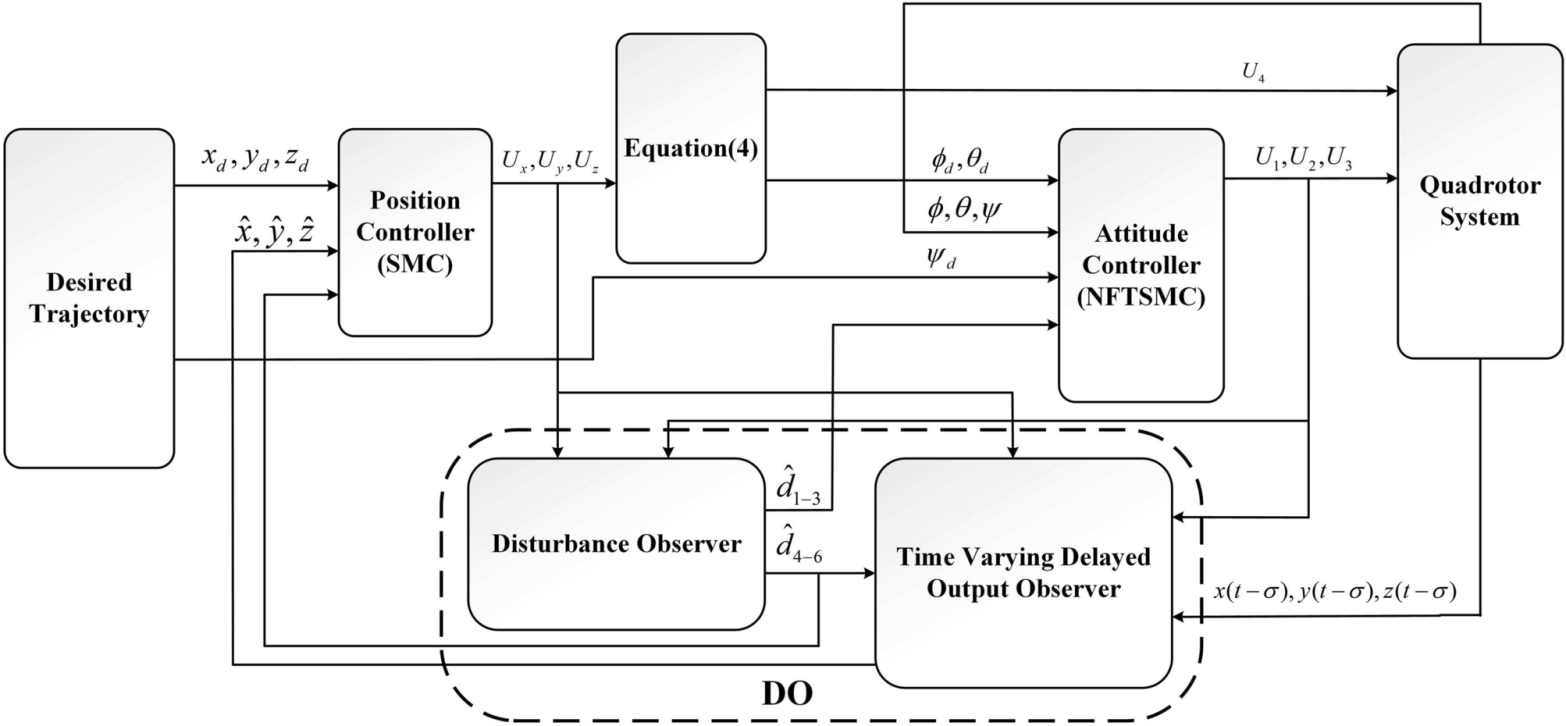
Control scheme.

The main objective of this paper is to design a control strategy such that the controlled QUAV has the disturbance-and-time delay-tolerant performance. According to the two subsystems, the corresponding control system is also divided into two control subsystem: inner loop and outer loop control subsystem. The control scheme is illustrated in Fig. 2, where the desired trajectory (${x_d}$, ${y_d}$, ${z_d}$ and ${\psi _d}$) is obtained by a signal emitter. Then, a SMC controller is designed to make the controlled QUAV track the position signals (${x_d}$, ${y_d}$ and ${z_d}$). and a NFTSMC controller is designed to guarantee the controlled angles tracking those desired angles. Moreover, a DO is given to estimate the unknown disturbances and time-varying delays.

### Design of the dual observers

#### Disturbance observer

The unknown disturbances, including internal uncertainties and external disturbances, can cause the instability or performance degradation of the QUAV system. In order to deal with these disturbances, a disturbance observer is proposed to estimate the actual values of the disturbances. According to the reference^29^, the disturbance observers for the attitude and position subsystems are given in Equations (7) and (8), respectively.

7 $$\begin{aligned}&\left\{ \begin{aligned}&{{\dot{Z}}_i} = {L_i}( - f({X_{2i}}) - {b_i}{u_i}) - {L_i}{{{\hat{d}}}_i}\\&{{{\hat{d}}}_i} = {Z_i} + {L_i}{x_{2i}}, \hspace{1em}i = 1,2,3\\ \end{aligned} \right. \end{aligned}$$

8 $$\begin{aligned}&\left\{ \begin{aligned}&{{\dot{Z}}_j} = {L_j}({g_j} - f({X_{2j}}) - {u_j}) - {L_j}{{{\hat{d}}}_j}\\&{{{\hat{d}}}_j} = {Z_j} + {L_j}{x_{2j}},\hspace{1em}j = 4,5,6 \end{aligned} \right. \end{aligned}$$

where ${Z_i}$ , ${Z_j}$ are the auxiliary reference variables. ${L_i},{L_j} \in {R^ + }$ are the observer gains. ${{\hat{d}}_i}$ and ${{\hat{d}}_j}$ are the estimated values of ${d_i}$ and ${d_j}$,respectively.

Generally, there is no priori information about the differential of the disturbance ${d_i}$ . Fortunately, compared with the dynamic properties of the observer, the variation of ${d_i}$ is much slow. Thus, we can assume that

9 $$\begin{aligned} {\dot{d}_i} = 0 \end{aligned}$$

Define the estimation errors of the observer as:

10 $$\begin{aligned} {{\tilde{d}}_i} = {d_i} - {{\hat{d}}_i} \end{aligned}$$

Substituting equation (5), (7) and (9) into the derivative of ${{\tilde{d}}_i}$ yields:

11 $$\begin{aligned} \begin{aligned} \dot{{\tilde{d}}}_i&= \dot{d}_i - \dot{{\hat{d}}}_i \\&= - \dot{Z}_i - L_i \dot{x}_{2i} \\&= - [L_i(- f(X_{2i}) - b_i u_i) - L_i {\hat{d}}_i] - L_i \dot{x}_{2i} \\&= - [L_i(- f(X_{2i}) - b_i u_i) - L_i {\hat{d}}_i] - L_i [f(X_{2i}) + b_i u_i + d_i] \\&= L_i ({\hat{d}}_i - d_i) \\&= - L_i {\tilde{d}}_i \end{aligned} \end{aligned}$$

Thus, the observer error equation can be written as:

12 $$\begin{aligned} {{\dot{{\tilde{d}}}}_i} + {L_i}{{\tilde{d}}_i} = 0 \end{aligned}$$

Solving the equation (12) yields:

13 $$\begin{aligned} {{\tilde{d}}_i}(t) = {{\tilde{d}}_i}({t_0}){e^{ - {L_i}t}} \end{aligned}$$

Similarly, we obtain:

14 $$\begin{aligned} {{\tilde{d}}_j}(t) = {{\tilde{d}}_j}({t_0}){e^{ - {L_j}t}} \end{aligned}$$

From Eqs. (13) and (14), it can be seen that the observer is asymptotically stable and their rate of convergence depends on the values of the parameters ${L_i}$ and ${L_j}$. Morover, the estimates ${{\hat{d}}_i}$ and ${{\hat{d}}_j}$ are exponentially approximated to the disturbances ${d_i}$ and ${d_j}$.

#### Time-varying delay observer

Besides the disturbances, measurement delays caused by low-quality GPS sensors also wreak havoc on the stability of the position subsystem of the QUAVs. Moreover, the delayed signals are usually time-varying, and lead to a significant degradation of control performance. Therefore, it is necessary to design a time-varying delay observer for the position subsystem. Considering the time-varying delayed signals and unknown disturbances, the dynamical model of the QUAV can be expressed as follows:

15 $$\begin{aligned} \dot{x}(t) = Fx(t) + G(x_j,t,{u_j}) + H{d_j},\hspace{1em}j = 4,5,6 \end{aligned}$$

where ${x_j} = {[{x_{2j - 1}},{x_{2j}}]^T}$. $G(x_j,t,{u_j}) = H\chi (x_j,t,{u_j})$ is a Lipschitz function. $F = [0,1;0,0]$, $H = {[0,1]^T}$.

The measured output signal is represented as:

16 $$\begin{aligned} {\bar{y}}(t) = Cx(t - \sigma (t)) \end{aligned}$$

where $C = [1,0]$, and $\sigma (t)$ is the time-varying delay, which is usually considered to be bounded, i.e. $\sigma (t) \in [0,\nabla ]$. $\nabla$ is a positive constant.

For the system (15), the time-varying delay observer is proposed as follows:

17 $$\begin{aligned} {\dot{\hat{x}}(t) }=&F{\hat{x}}(t) + G({\hat{x}}_j,t,{u_j}) + K[\bar{y}(t) - C{\hat{x}}(t - \sigma (t))] + H{{\hat{d}}_j},\hspace{1em}j = 4,5,6 \end{aligned}$$

For the second-order systems, the observer is specifically represented as follows:

18 $$\begin{aligned} \left\{ \begin{aligned}&{{\dot{\hat{x}}}_{2j - 1}} = {{{\hat{x}}}_{2j}} + {k_{1j}}({x_{2j - 1}}(t - \sigma (t)) - {{{\hat{x}}}_{2j - 1}}(t - \sigma (t)))\\&{{\dot{\hat{x}}}_{2j}} = \chi ({\hat{x}}_j,t,{u_j}) + {k_{2j}}({x_{2j - 1}}(t - \sigma (t))-{{{\hat{x}}}_{2j-1}}(t - \sigma (t))) + {{{\hat{d}}}_j},\hspace{1em}j = 4,5,6\\ \end{aligned} \right. \end{aligned}$$

where ${\hat{x}}$ is the estimation of *x*, and ${\hat{x}}(t - \sigma (t))$ is the estimated delayed state signal. $K = {[{k_{1j}},{k_{2j}}]^T}$ is the observer gain matrix and must be designed to satisfy $F - KC$ Hurwitz. To make the $F - KC$ satisfies the Hurwitz condition, we select :

19 $$\begin{aligned} \begin{aligned} M = F - KC = \left[ {\begin{array}{*{20}{c}} 0& 1\\ 0& 0 \end{array}} \right] - \left[ {\begin{array}{*{20}{c}} {{k_{1j}}}\\ {{k_{2j}}} \end{array}} \right] \left[ {\begin{array}{*{20}{c}} 1&0 \end{array}} \right] = \left[ {\begin{array}{*{20}{c}} { - {k_{1j}}}& 1\\ { - {k_{2j}}}& 0 \end{array}} \right] \end{aligned} \end{aligned}$$

The characteristic equation is:

20 $$\begin{aligned} \begin{aligned} \left| {\lambda I - M} \right| = \left| {\begin{array}{*{20}{c}} {\lambda + {k_{1j}}}& 1\\ { - {k_{2j}}}& \lambda \end{array}} \right| = {\lambda ^2} + {k_{1j}}\lambda + {k_{2j}} = 0 \end{aligned} \end{aligned}$$

Comparing Equation (20) with ${(\lambda + {k_n})^2} = {\lambda ^2} + 2{k_n}\lambda + {k_n}^2 = 0$, $({k_n} > 0,n = x,y,z)$, we get:

$$\begin{aligned} \left\{ \begin{aligned} {k_{1j}} = 2{k_n}\\ {k_{2j}} = {k_n}^2 \end{aligned} \right. \end{aligned}$$

By choosing a suitable ${k_n}$, we can ensure that the *M* matrix satisfies the Hurwitz properties.

The goal is to make the estimated signal track the actual signal when $t \rightarrow \infty$, i.e. ${\hat{x}}(t) \rightarrow x(t)$ as $t \rightarrow \infty$. Defining the estimation error as ${\tilde{x}}(t) = x(t) - {\hat{x}}(t)$, and subtracting Equation (17) from Equation (15) yields:

21 $$\begin{aligned} \begin{aligned} {\dot{\tilde{x}}(t) }= F{\tilde{x}}(t) + \tilde{G}({\tilde{x}}_j,t,x_j) + N{\tilde{x}}(t - \sigma (t)) + H{\tilde{d}_j},\hspace{1em}j = 4,5,6 \end{aligned} \end{aligned}$$

where $${\tilde{G}}({\tilde{x}}_j,t,x_j) = G(x_j,t,{u_j}) - G({\hat{x}}_j,t,{u_j})$$ and $$N=-KC$$.

To eliminate the effect of ${{\tilde{d}}_j}$ on the system (21), a large L in the disturbance observer (8) is needed to ensure that ${{\hat{d}}}$ converges to the real value faster than that of the ${{\hat{x}}}$ in the observer (17). Thus, equation (21) can be rewritten as:

22 $$\begin{aligned} \begin{aligned} {\dot{\tilde{x}}(t)} =&F{\tilde{x}}(t) + {\tilde{G}}({\tilde{x}}_j,t,x_j) + N{\tilde{x}}(t - \sigma (t))\\ \mathrm{ } =&(F + N){\tilde{x}}(t) + {\tilde{G}}({\tilde{x}}_j,t,x_j) - N({\tilde{x}}(t)- {\tilde{x}}(t - \sigma (t))) \end{aligned} \end{aligned}$$

For $t > \sigma (t)$, ${\tilde{x}}(t) - {\tilde{x}}(t - \sigma (t) ) = \int _{t - \sigma (t) }^t {\dot{{\tilde{x}}}(\xi )} d\xi$, let $\rho = \xi - t$, we get:

23 $$\begin{aligned} \begin{aligned} {\tilde{x}}(t) - {\tilde{x}}(t - \sigma (t) ) =&\int _{ - \sigma }^0 {\dot{\tilde{x}}(\rho + t)} d\rho \\ \mathrm{ } =&\int _{ - \sigma }^0 {F{\tilde{x}}(\rho + t)} + N{\tilde{x}}(\rho + t - \sigma (t)) + {\tilde{G}}(\tilde{x}_j(\rho + t),\rho + t,x_j(\rho + t))d\rho \end{aligned} \end{aligned}$$

Denoting ${{\tilde{x}}_t}(\rho ) = {\tilde{x}}_j(\rho + t)$ and ${\tilde{x}_t}(\rho - \sigma (t) ) = {\tilde{x}}(\rho + t - \sigma (t) )$, and substituting equation (23) into equation (22), we obtain:

24 $$\begin{aligned} \begin{aligned} {\dot{\tilde{ x}}(t)} =&(F + N){\tilde{x}}(t) + {\tilde{G}}({\tilde{x}}_j,t,x_j) - N(\int _{ - \sigma }^0 {F{{\tilde{x}}_t}(t)} + N{{\tilde{x}}_t}(\rho - \sigma (t)) + {\tilde{G}}({\tilde{x}_t}(\rho ),\rho + t,{x_t}(\rho ))d\rho ) \end{aligned} \end{aligned}$$

Define ${\mu _1} = {\tilde{G}}({\tilde{x}}_j,t,x_j)$ and ${\mu _2} = - N(\int _{ - \sigma }^0 {F{{{\tilde{x}}}_t}(t)} + N{{\tilde{x}}_t}(\rho - \sigma (t)) + {\tilde{G}}({{\tilde{x}}_t}(\rho ),\rho + t,{x_t}(\rho ))d\rho )$, then equation (24) can be simplified as follows:

25 $$\begin{aligned} \begin{aligned} {\dot{\tilde{ x}}(t)} = (F + N){\tilde{x}}(t) + {\mu _1} + {\mu _2} \end{aligned} \end{aligned}$$

##### Lemma 1

Reference^24^: $V(\varepsilon (t))$ is a Lyapunov-Razumikhin candidate function such that the following inequality condition $V({\varepsilon _t}(\rho )) \le pV$ holds, where ${\varepsilon _t}(\rho ) = \varepsilon (t + \rho )$, $\forall \rho \in [ - 2\Delta ,0]$ and $p > 1$ can be chosen arbitrarily close to 1. If it satisfies that $\dot{V}(\varepsilon (t)) \le - \vartheta \left\| {\varepsilon (t)} \right\|$, with $\vartheta :\mathbb {R} ^ { + } \rightarrow \mathbb {R} ^ { + }$ continuous, positive definite and nondecreasing, then $\varepsilon (t)$ is uniformly asymptotically convergence to 0.

The consistent asymptotic stability of equation (25) can be guaranteed by Lemma 1, and a more detailed proof is given in^24^. Thus, it confirms the asymptotic stability of equation (21) as well as ${\lim _{t \rightarrow \infty }}\left\| {\tilde{x}(t)} \right\| = 0$.

### Design of the attitude controller

Attitude control, as the inner loop control, is the main control of QUAVs and it directly affects the stability of the outer loop control (position control). Therefore, it is necessary to design a fast converging sliding mode controller for the attitude loop.

The desired angles ${\phi _d}$ and ${\theta _d}$ are given in equation (4) and the desired yaw ${\psi _d}$ is an input. Let the desired angles be ${x_{(2i - 1)d}}$, $i = 1,2,3$. Then, the attitude tracking errors and their derivative are expressed as follows:

26 $$\begin{aligned}&{e_i} = {x_{2i - 1}} - {x_{(2i - 1)d}} \end{aligned}$$

27 $$\begin{aligned}&{\dot{e}_i} = {\dot{x}_{2i - 1}} - {\dot{x}_{(2i - 1)d}} \end{aligned}$$

For the attitude subsystem (5), the new sliding surfaces and their derivative are given by:

28 $$\begin{aligned}&{S_i} = {\dot{e}_i} + {\lambda _i}{\left| {{e_i}} \right| ^{{\gamma _i}}}sign({e_i}) \end{aligned}$$

29 $$\begin{aligned}&{\dot{S}_i} = {\ddot{e}_i} + {\lambda _i}{\gamma _i}{\left| {{e_i}} \right| ^{{\gamma _i} - 1}}{\dot{e}_i} \end{aligned}$$

where ${\lambda _i} > 0$ , and ${\gamma _i}$ is defined by:

30 $$\begin{aligned} {\gamma _i} = \left\{ \begin{aligned}&\frac{{{p_i}}}{{{q_i}}},&\mathrm{ }if\mathrm{ }\varsigma< \left| {{e_i}} \right| < 1\\&1,&\mathrm{ }else \end{aligned} \right. \end{aligned}$$

where ${p_i}$, ${q_i}$ are positive odd numbers and $0< \frac{{{p_i}}}{{{q_i}}} < 1$. $\varsigma$ is a small positive constant.

Equation (28) is a new nonsingular fast terminal sliding mode surface that dynamically switches between linear sliding mode surface and terminal sliding mode surface by means of the exponential term ${\gamma _i}$. When $\left| {{e_i}} \right|$ is away from zero, it is set as a linear slide mode surface, and when $\left| {{e_i}} \right|$ is close to zero, it is switched to a terminal slide mode surface. The reason for this design is that the terminal sliding mode surface does not converge as fast as the linear sliding mode surface when $\left| {{e_i}} \right|$ is far from zero, while it converges faster than the linear sliding mode surface when $\left| {{e_i}} \right|$ is close to zero^30^. In addition, considering equation (29), $\varsigma$ is chosen to be a very small positive constant to avoid the singularity when ${e_i} = 0$ and ${\dot{e}_i} \ne 0$.

Therefore, for the attitude subsystem (5), the disturbance-observer-based sliding mode control laws are designed as:

31 $$\begin{aligned} \begin{aligned} {u_i} =&{b_i}^{ - 1}({\ddot{x}_{(2i - 1)d}} - f({X_{2i}}) - {\lambda _i}{\gamma _i}{\left| {{e_i}} \right| ^{{\gamma _i} - 1}}{\dot{e}_i}- {{\hat{d}}_i} - {K_i}{S_i} - {\eta _i}sign({S_i})), \hspace{1em} i= 1,2,3 \end{aligned} \end{aligned}$$

where ${K_i}$ and ${\eta _i}$ are positive constants.

#### Lemma 2

Reference^31^: For $V:[0,\infty ) \in R$, the solution of the inequality equation $\dot{V} \le - \alpha V + f$, $\forall t \ge {t_0} \ge 0$ is as follows:

32 $$\begin{aligned} \begin{aligned} V \le {e^{ - \alpha (t - {t_0})}}V({t_0}) + \int _{{t_0}}^t {{e^{ - \alpha (t - \tau )}}} f(\tau )d\tau \end{aligned} \end{aligned}$$

where $\alpha$ is an arbitrary constant.

#### Theorem 1

In attitude subsystems with unknown disturbances, the proposed disturbance-observer-based sliding mode control laws (31) ensures that the closed-loop subsystem is asymptotically stable and the tracking error converges to zero quickly.

#### Proof

Select the Lyapunov candidate function as follows:

33 $$\begin{aligned} \begin{aligned} {V_a} = \sum \limits _{i = 1}^3 {(\frac{1}{2}{S_i}^2 + \frac{1}{2}{{{\tilde{d}}}_i}^2)} \end{aligned} \end{aligned}$$

The derivative of ${V_a}$ is given by:

34 $$\begin{aligned} \begin{aligned} {{\dot{V}}_a} =&\sum \limits _{i = 1}^3 {({S_i}{{\dot{S}}_i} + {{{\tilde{d}}}_i}{{\dot{{\tilde{d}}}}_i})} \\ =&\sum \limits _{i = 1}^3 {({S_i}({{\ddot{e}}_i} + {\lambda _i}{\gamma _i}{{\left| {{e_i}} \right| }^{{\gamma _i} - 1}}{{\dot{e}}_i}) + {{{\tilde{d}}}_i}{{\dot{{\tilde{d}}}}_i})} \\ =&\sum \limits _{i = 1}^3 {({S_i}({{\ddot{x}}_{2i - 1}} - {{\ddot{x}}_{(2i - 1)d}} + {\lambda _i}{\gamma _i}{{\left| {{e_i}} \right| }^{{\gamma _i} - 1}}{{\dot{e}}_i}) + {{{\tilde{d}}}_i}{{\dot{{\tilde{d}}}}_i})} \\ =&\sum \limits _{i = 1}^3 {({S_i}(f({X_{2i}}) + {b_i}{u_i} + {d_i} - {{\ddot{x}}_{(2i - 1)d}} + {\lambda _i}{\gamma _i}{{\left| {{e_i}} \right| }^{{\gamma _i} - 1}}{{\dot{e}}_i}) + {{{\tilde{d}}}_i}{{\dot{{\tilde{d}}}}_i})} \end{aligned} \end{aligned}$$

Substituting Eqs. (11) and (31) into equation (34), we get:

35 $$\begin{aligned} \begin{aligned} {{\dot{V}}_a}&= \sum \limits _{i = 1}^3 {({S_i}({{{\tilde{d}}}_i} - {K_i}{S_i} - {\eta _i}sign({S_i})) + {{{\tilde{d}}}_i}( - {L_i}{{{\tilde{d}}}_i}))} \\&= \sum \limits _{i = 1}^3 {({{{\tilde{d}}}_i}{S_i} - {K_i}{S_i}^2 - {\eta _i}\left| {{S_i}} \right| - {L_i}{{{\tilde{d}}}_i}^2)} \\&\le \sum \limits _{i = 1}^3 {(\left| {{{{\tilde{d}}}_i}} \right| \left| {{S_i}} \right| - {K_i}{S_i}^2 - {\eta _i}\left| {{S_i}} \right| - {L_i}{{{\tilde{d}}}_i}^2)} \\&= \sum \limits _{i = 1}^3 {((\left| {{{{\tilde{d}}}_i}} \right| - {\eta _i})\left| {{S_i}} \right| - {K_i}{S_i}^2 - {L_i}{{{\tilde{d}}}_i}^2)} \\&\le \sum \limits _{i = 1}^3 {( - {K_i}{S_i}^2 - {L_i}{{{\tilde{d}}}_i}^2)} \\&\le \sum \limits _{i = 1}^3 {( - {K_0}(\frac{1}{2}{S_i}^2 + \frac{1}{2}{{{\tilde{d}}}_i}^2))} \mathrm{{ = }} - {K_0}{V_a} \end{aligned} \end{aligned}$$

where $${\eta _i} \ge {\left| {{{{\tilde{d}}}_i}} \right| _{\max }}$$ and $${K_0} = 2\min \left\{ {\left. {{K_i},{L_i}} \right\} } \right.$$.

Using Lemma 2, the solution of inequality ${\dot{V}_a} \le - {K_0}{V_a}$ is obtained as:

36 $$\begin{aligned} \begin{aligned} {V_a}(t) \le {e^{ - {K_0}(t - {t_0})}}{V_a}({t_0}) \end{aligned} \end{aligned}$$

It follows that the control system is exponential convergence, and the convergence accuracy depends on the parameter ${K_0}$. Therefore, the attitude subsystem is asymptotically stable under control law (31), i.e. $\mathop {\lim }\limits _{t \rightarrow \infty } {S_i} = 0$, $\mathop {\lim }\limits _{t \rightarrow \infty } {\tilde{d}_i} = 0$, $\mathop {\lim }\limits _{t \rightarrow \infty } {e_i} = 0$ ($i = 1,2,3$).

In order to reduce the chattering of the controllers, the *sign* function is replaced by a hyperbolic tangent function in this paper. The hyperbolic tangent function is expressed as:

$$\begin{aligned} \tanh (\frac{s}{\Delta }) = \frac{{{e^{s/\Delta }} - {e^{ - s/\Delta }}}}{{{e^{s/\Delta }} + {e^{ - s/\Delta }}}} \end{aligned}$$

where $\Delta$ is a positive constant.

### Design of the position controller

The control system is composed of two parts: the inner and outer loops.The outer loop convergence rate should be lower than the inner loop convergence rate to ensure the stability of the whole closed-loop system^32^. Hence, a relatively slow sliding mode controller, which is combined with a DO, is adopted to control the position subsystem, and counteract the unknown disturbances and time-varying delays.

Setting the desired position as ${x_{(2j - 1)d}}$, $j= 4,5,6$, the position tracking errors and their derivative can be defined as:

37 $$\begin{aligned}&\begin{aligned} {e_j} = {x_{2j - 1}} - {x_{(2j - 1)d}} \end{aligned} \end{aligned}$$

38 $$\begin{aligned}&\begin{aligned} {\dot{e}_j} = {\dot{x}_{2j - 1}} - {\dot{x}_{(2j - 1)d}} \end{aligned} \end{aligned}$$

The sliding surface is selected as:

39 $$\begin{aligned} \begin{aligned} {S_j} = {\dot{e}_j} + {\lambda _j}{e_j} \end{aligned} \end{aligned}$$

Substituting equation (6) into the derivative of (39), one yields:

40 $$\begin{aligned} \begin{aligned} {{\dot{S}}_j}&= {{\ddot{e}}_j} + {\lambda _j}{{\dot{e}}_j}\\&= {{\ddot{x}}_{2j - 1}} - {{\ddot{x}}_{(2j - 1)d}} + {\lambda _j}{{\dot{e}}_j}\\&= f({X_{2j}}) + {u_j} - {g_j} + {d_j} - {{\ddot{x}}_{(2j - 1)d}} + {\lambda _j}{{\dot{e}}_j} \end{aligned} \end{aligned}$$

Let ${\dot{S}_j} = 0$, considering observer (8) and (17), the DO-based sliding mode control laws can be given as follows:

41 $$\begin{aligned} \begin{aligned} {u_j} = {\ddot{x}_{(2j - 1)d}} - f({{\hat{X}}_{2j}}) - {\lambda _j}{\dot{\hat{ e_j}}} + {g_j} - {{\hat{d}}_j} - {K_j}{{\hat{S}}_j},\hspace{1em} j = 4,5,6 \end{aligned} \end{aligned}$$

where ${[f({{\hat{X}}_8}),f({{\hat{X}}_{10}}),f({{\hat{X}}_{12}})]^T} = {[ - {k_4}{{\hat{x}}_8}/m, - {k_5}{{\hat{x}}_{10}}/m, - {k_6}{{\hat{x}}_{12}}/m]^T}$, ${{\hat{e}}_j} = {{\hat{x}}_{2j - 1}} - {x_{(2j - 1)d}}$, ${\dot{\hat{e_j}} }= {{\hat{x}}_{2j}} - {\dot{x}_{(2j - 1)d}}$, ${{\hat{S}}_j} = {\dot{\hat{e_j}}} + {\lambda _j}{{\hat{e}}_j}$; ${K_j}$ and ${\eta _j}$ are positive constants.

#### Theorem 2

Considering a position subsystem with the unknown disturbances and time-varying delays, the proposed DO-based sliding mode control laws ensure that the closed-loop subsystem is rapidly convergent and stable. 

#### Proof

The Lyapunov function is defined as:

42 $$\begin{aligned} \begin{aligned} {V_p} = \sum \limits _{j = 4}^6 {(\frac{1}{2}{S_j}^2 + \frac{1}{2}{{{\tilde{d}}}_j}^2)} \end{aligned} \end{aligned}$$

Taking the derivative of ${V_p}$, we obtain:

43 $$\begin{aligned} \begin{aligned} {\dot{V}_p} = \sum \limits _{j = 4}^6 {({S_j}{{\dot{S}}_j} + {{{\tilde{d}}}_j}{{\dot{{\tilde{d}}}}_j})} \end{aligned} \end{aligned}$$

Substituting controller (41) into the derivative of ${S_i}$, we obtain:

44 $$\begin{aligned} \begin{aligned} \sum \limits _{j = 4}^6 {{{\dot{S}}_j}} =&\sum \limits _{j = 4}^6 {({{\ddot{x}}_{2j - 1}} - {{\ddot{x}}_{(2j - 1)d}} + {\lambda _j}{{\dot{e}}_j})} \\ =&\sum \limits _{j = 4}^6 {(f({X_{2j}}) + {u_j} - {g_j} + {d_j} - {{\ddot{x}}_{(2j - 1)d}} + {\lambda _j}{{\dot{e}}_j})} \\ =&\sum \limits _{j = 4}^6 {(f({X_{2j}}) - f({{{\hat{X}}}_{2j}}) + {\lambda _j}{{\dot{e}}_j} - {\lambda _j}{{\dot{\hat{e}}}_j} + {d_j} - {{{\hat{d}}}_j} - {K_j}{{{\hat{S}}}_j})} \\ =&\sum \limits _{j = 4}^6 {( - \frac{{{k_j}{x_{2j}}}}{m} + \frac{{{k_j}{{{\hat{x}}}_{2j}}}}{m} + {\lambda _j}{{\dot{e}}_j} - {\lambda _j}{{\dot{\hat{e}}}_j} + {d_j} - {{{\hat{d}}}_j} - {K_j}{{{\hat{S}}}_j})} \\ =&\sum \limits _{j = 4}^6 {( - \frac{{{k_j}{{{\tilde{x}}}_{2j}}}}{m} + {\lambda _j}{{{\tilde{x}}}_{2j}} + {{{\tilde{d}}}_j} - {K_j}({S_j} - {{{\tilde{S}}}_j}))} \\ =&\sum \limits _{j = 4}^6 {(({\lambda _j} - \frac{{{k_j}}}{m}){{{\tilde{x}}}_{2j}} + {{{\tilde{d}}}_j} - {K_j}({S_j}- {\lambda _j}{{{\tilde{x}}}_{2j - 1}} - {{{\tilde{x}}}_{2j}}))} \\ =&\sum \limits _{j = 4}^6 {( - {K_j}{S_j} + ({\lambda _j} + {K_j} - \frac{{{k_j}}}{m}){{{\tilde{x}}}_{2j}} + {K_j}{\lambda _j}{{\tilde{x}}_{2j - 1}} + {{{\tilde{d}}}_j})} \end{aligned} \end{aligned}$$

where $${{\tilde{x}}_{2j - 1}} = {x_{2j - 1}} - {{\hat{x}}_{2j - 1}}$$, $${{\tilde{x}}_{2j}} = {x_{2j}} - {{\hat{x}}_{2j}}$$, $${{\tilde{e}}_j} = {e_j} - {{\hat{e}}_j} = {x_{2j - 1}} - {{\hat{x}}_{2j - 1}} = {{\tilde{x}}_{2j - 1}}$$, $${\tilde{{\dot{e}}}_j} = {\dot{e}_j} - {\dot{\hat{e}}_j} = {x_{2j}} - {{\hat{x}}_{2j}} = {{\tilde{x}}_{2j}}$$, $${{\tilde{S}}_j} = {S_j} - {{\hat{S}}_j} = {\lambda _j}{{\tilde{x}}_{2j - 1}} + {{\tilde{x}}_{2j}}$$.

Substituting equation (44) into equation (43), one yields:

45 $$\begin{aligned} \begin{aligned} {{\dot{V}}_p} =&\sum \limits _{j = 4}^6 {({S_j}{{\dot{S}}_j} + {{{\tilde{d}}}_j}{{\dot{{\tilde{d}}}}_j})} \\ =&\sum \limits _{j = 4}^6 {({S_j}( - {K_j}{S_j} + {K_j}{\lambda _j}{{{\tilde{x}}}_{2j - 1}} + ({\lambda _j} + {K_j} - \frac{{{k_j}}}{m}){{{\tilde{x}}}_{2j}}+ {{{\tilde{d}}}_j}) + {{{\tilde{d}}}_j}{{\dot{{\tilde{d}}}}_j})} \\ =&\sum \limits _{j = 4}^6 {( - {K_j}{S_j}^2 + {S_j}({K_j}{\lambda _j}{{{\tilde{x}}}_{2j - 1}} + ({\lambda _j} + {K_j} - \frac{{{k_j}}}{m}){{{\tilde{x}}}_{2j}})+ {S_j}{{{\tilde{d}}}_j} + {{{\tilde{d}}}_j}{{\dot{{\tilde{d}}}}_j})} \\ =&\sum \limits _{j = 4}^6 {( - {K_j}{S_j}^2 + {S_j}(O({{{\tilde{x}}}_{2j - 1}},{{{\tilde{x}}}_{2j}})) + {S_j}{{{\tilde{d}}}_j} + {{\tilde{d}}_j}{{\dot{{\tilde{d}}}}_j})} \end{aligned} \end{aligned}$$

where $$O({{\tilde{x}}_{2j - 1}},{{\tilde{x}}_{2j}}) = {K_j}{\lambda _j}{{\tilde{x}}_{2j - 1}} + ({\lambda _j} + {K_j} - \frac{{{k_j}}}{m}){{\tilde{x}}_{2j}}$$.

The time-varying delay observer (17) is asymptotically convergent, and $O({{\tilde{x}}_{2j - 1}},{{\tilde{x}}_{2j}}) \le {O_{\max }}$, thus

46 $$\begin{aligned} \begin{aligned} {{\dot{V}}_p} =&\sum \limits _{j = 4}^6 {( - {K_j}{S_j}^2 + {S_j}(O({{{\tilde{x}}}_{2j - 1}},{{{\tilde{x}}}_{2j}})) + {S_j}{{{\tilde{d}}}_j} + {{{\tilde{d}}}_j}{{\dot{\tilde{d}}}_j})} \\ \le&\sum \limits _{j = 4}^6 {( - {K_j}{S_j}^2 + {S_j}{O_{\max }} + {S_j}{{{\tilde{d}}}_j} + {{{\tilde{d}}}_j}{{\dot{\tilde{d}}}_j})} \\ \le&\sum \limits _{j = 4}^6 {( - {K_j}{S_j}^2 + \frac{1}{2}({S_j}^2 + O_{\max }^2) + \frac{1}{2}({S_j}^2 + {{{\tilde{d}}}_j}^2) - {L_j}{{{\tilde{d}}}_j}^2)} \\ =&\sum \limits _{j = 4}^6 {(\frac{1}{2}O_{\max }^2 - ({K_j} - 1){S_j}^2 - ({L_j} - \frac{1}{2}){{{\tilde{d}}}_j}^2)} \\ \le&\sum \limits _{j = 4}^6 {( - {\eta _0}(\frac{1}{2}{S_j}^2 + \frac{1}{2}{{{\tilde{d}}}_j}^2) + \frac{1}{2}O_{\max }^2)} \\ =&- {\eta _0}{V_p} + {O_{sum}} \end{aligned} \end{aligned}$$

where $${\eta _0} = 2\min \{ ({K_j} - 1),({L_j} - \frac{1}{2})\}$$, $${O_{sum}} = \frac{3}{2}O_{\max }^2$$.

According to Lemma 2, we can obtain

47 $$\begin{aligned} \begin{aligned} {V_p}(t) \le&{e^{ - {\eta _0}(t - {t_0})}}{V_p}({t_0}) + {O_{sum}}\int _{{t_0}}^t {{e^{ - {\eta _0}(t - \tau )}}} d\tau \\ =&{e^{ - {\eta _0}(t - {t_0})}}{V_p}({t_0}) + {e^{ - {\eta _0}t}}{O_{sum}}\int _{{t_0}}^t {{e^{{\eta _0}\tau )}}} d\tau \\ =&{e^{ - {\eta _0}(t - {t_0})}}{V_p}({t_0}) + \frac{{{O_{sum}}}}{{{\eta _0}}}(1 - {e^{ - {\eta _0}(t - {t_0})}}) \end{aligned} \end{aligned}$$

From the inequality (47), it has $\mathop {\lim }\limits _{t \rightarrow \infty } {V_p}(t) = \frac{{{O_{sum}}}}{{{\eta _0}}}$, that is, the tracking error convergence value depends on the values of ${O_{sum}}$ and ${\eta _0}$. If ${\eta _0}$ is large enough, it has $\mathop {\lim }\limits _{t \rightarrow \infty } {S_j} = 0$, $\mathop {\lim }\limits _{t \rightarrow \infty } {\dot{e}_j} = 0$, $\mathop {\lim }\limits _{t \rightarrow \infty } {e_j} = 0$ ($j = 4,5,6$), when ${O_{sum}}\rightarrow 0$.

## Numerical simulations

### Example 1

The performance of the control scheme is evaluated by the trajectory tracking of a QUAV with unknown disturbances and time-varying delays. The physical parameters of the QUAV are shown in Table 1 and the parameters of the controller are given in Table 2.

**Table 1:** The physical parameters of the QUAV.

Parameter	Value	Unit
*m*	0.48	*kg*
*l*	0.2	*m*
${I_1}$	$3.8 \times {10^{ - 3}}$	$kg \cdot {m^2}$
${I_2}$	$3.8 \times {10^{ - 3}}$	$kg \cdot {m^2}$
${I_3}$	$7.6 \times {10^{ - 3}}$	$kg \cdot {m^2}$
${k_r}(r = 1,2, \cdots 6)$	$5.6 \times {10^{ - 4}}$	*N*/*m*/*s*
*g*	9.8	$m/{s^2}$

**Table 2:** The parameters of the controller.

Parameter	Value	Parameter	Value
${p_1},{p_2},{p_3}$	5	${L_1},{L_2},{L_3}$	50
${q_1},{q_2},{q_3}$	9	${L_4},{L_5},{L_6}$	60
$\varsigma$	0.01	${k_x},{k_y},{k_z}$	1
${\lambda _1},{\lambda _2}$	48	${K_1},{K_2},{K_3}$	50
${\lambda _3}$	8	${K_4},{K_5},{K_6}$	5
${\lambda _4},{\lambda _5}$	5	${\eta _1},{\eta _2},{\eta _3}$	0.1
${\lambda _6}$	4	$\Delta$	0.01

In order to facilitate the simulation, the circular shape reference trajectory and the yaw angle are designed as following:

$$\begin{aligned} \left\{ \begin{aligned} {x_d}&= \frac{1}{2}\cos (\frac{t}{2})\\ {y_d}&= \frac{1}{2}\sin (\frac{t}{2})\\ {z_d}&= 1\\ {\psi _d}&= \frac{\pi }{3} \end{aligned} \right. \end{aligned}$$

**Fig. 3:**
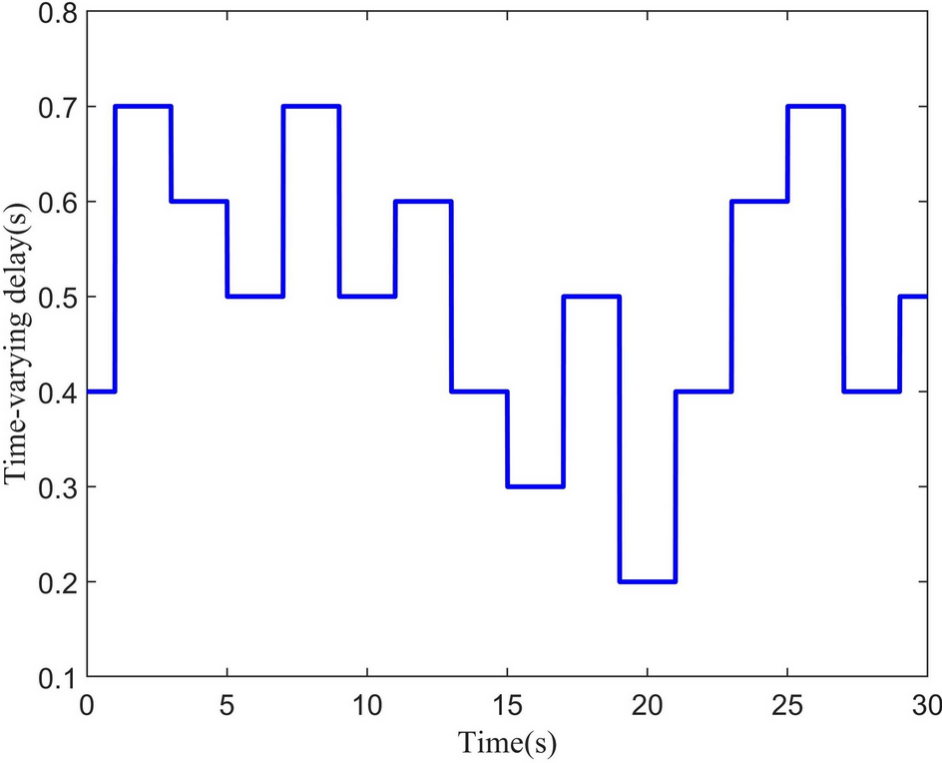
Time-varying $\sigma (t)$ delay in range [0, 1].

As shown in Fig. 3, the time-varying delay $\sigma (t)$ is chosen randomly from the range [0, 1]. The disturbances are considered as follows:

$$\begin{aligned} \left\{ \begin{aligned} {d_i}&= 0.1\sin (t), \hspace{1em}i = 1,2,3 \\ {d_j}&= 0.1\cos (t), \hspace{1em}j = 4,5,6 \end{aligned} \right. \end{aligned}$$

**Fig. 4:**
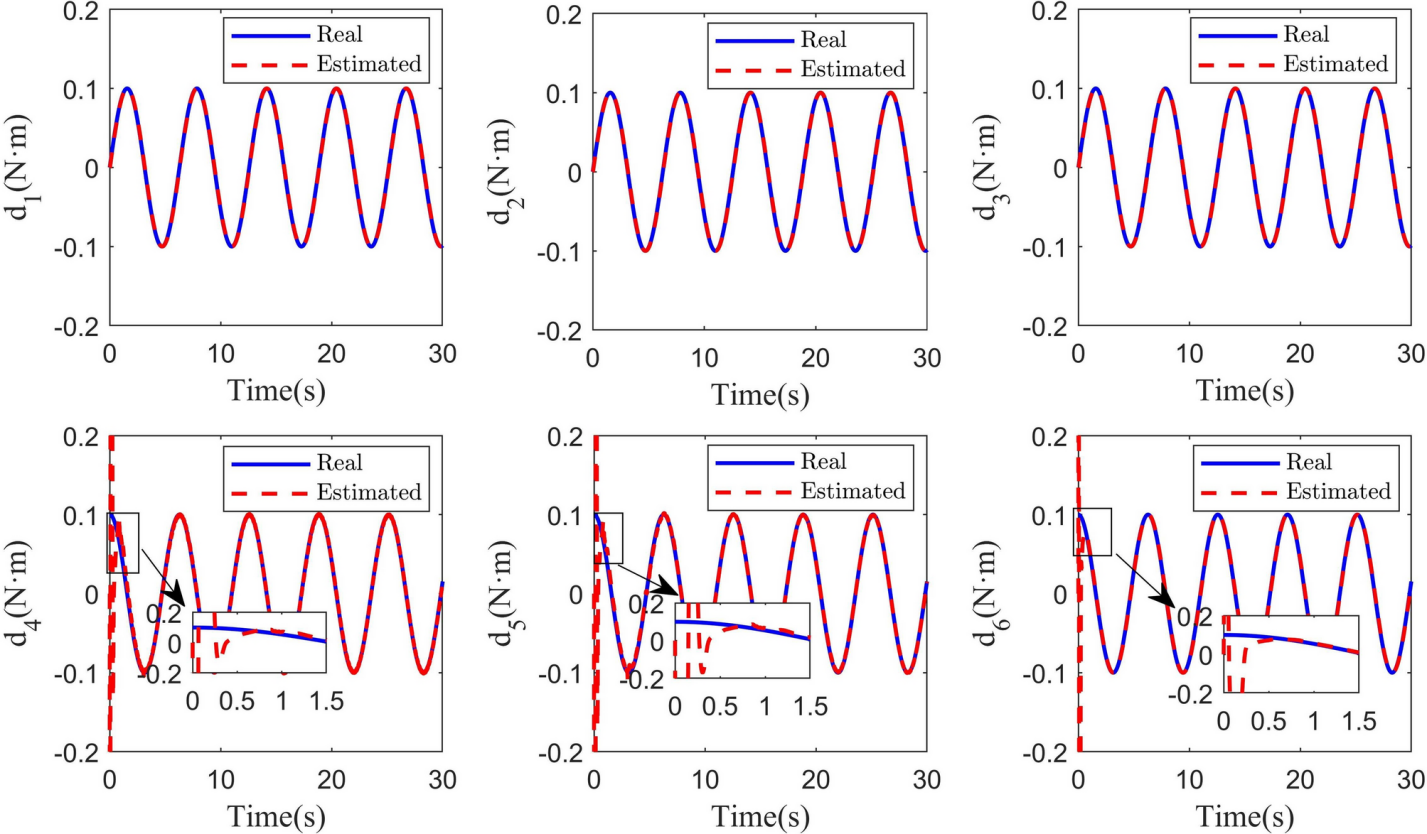
Real and estimated values of ${d_s}(s = 1,2, \cdots ,6)$.

**Fig. 5:**
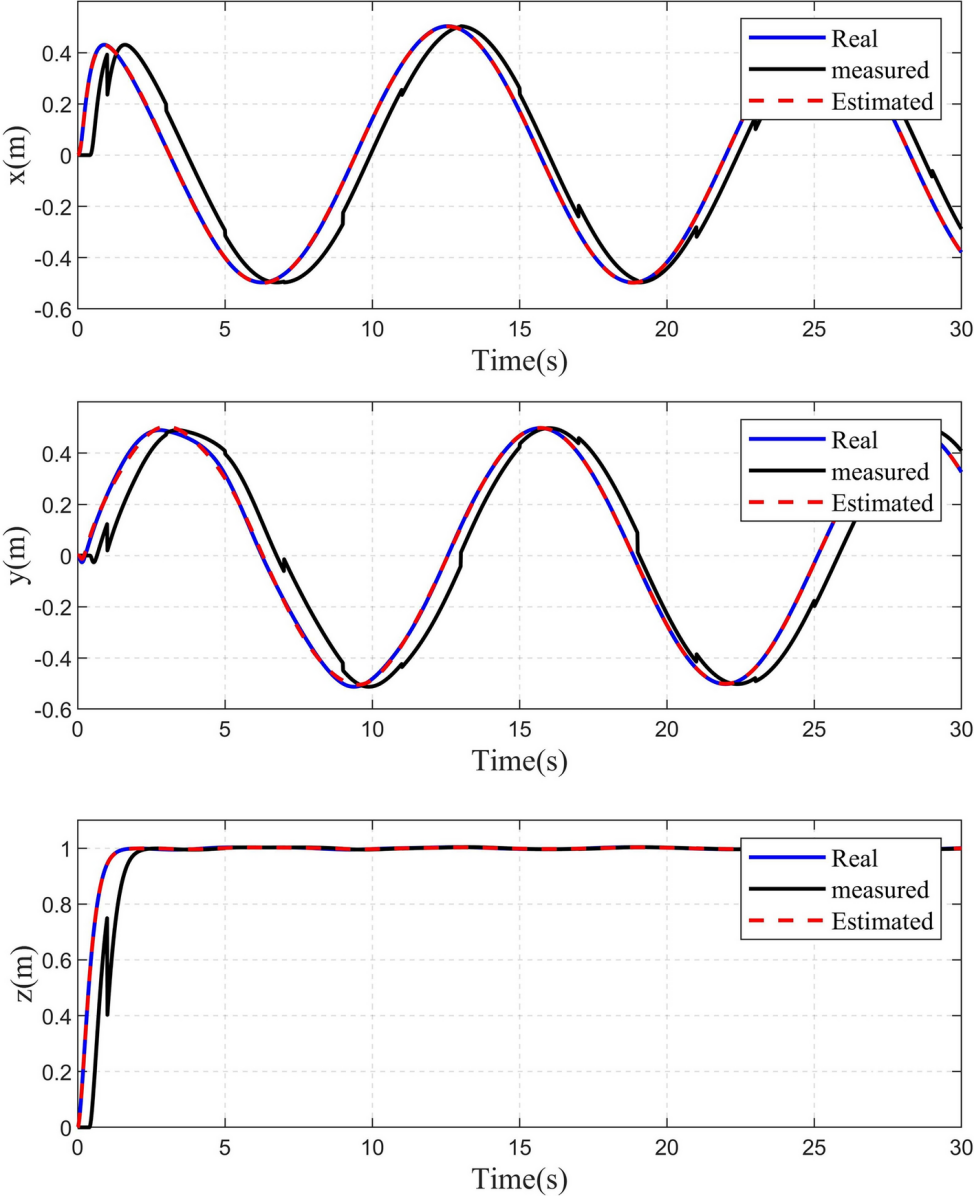
Real, measured and estimated values of x, y and z.

**Fig. 6:**
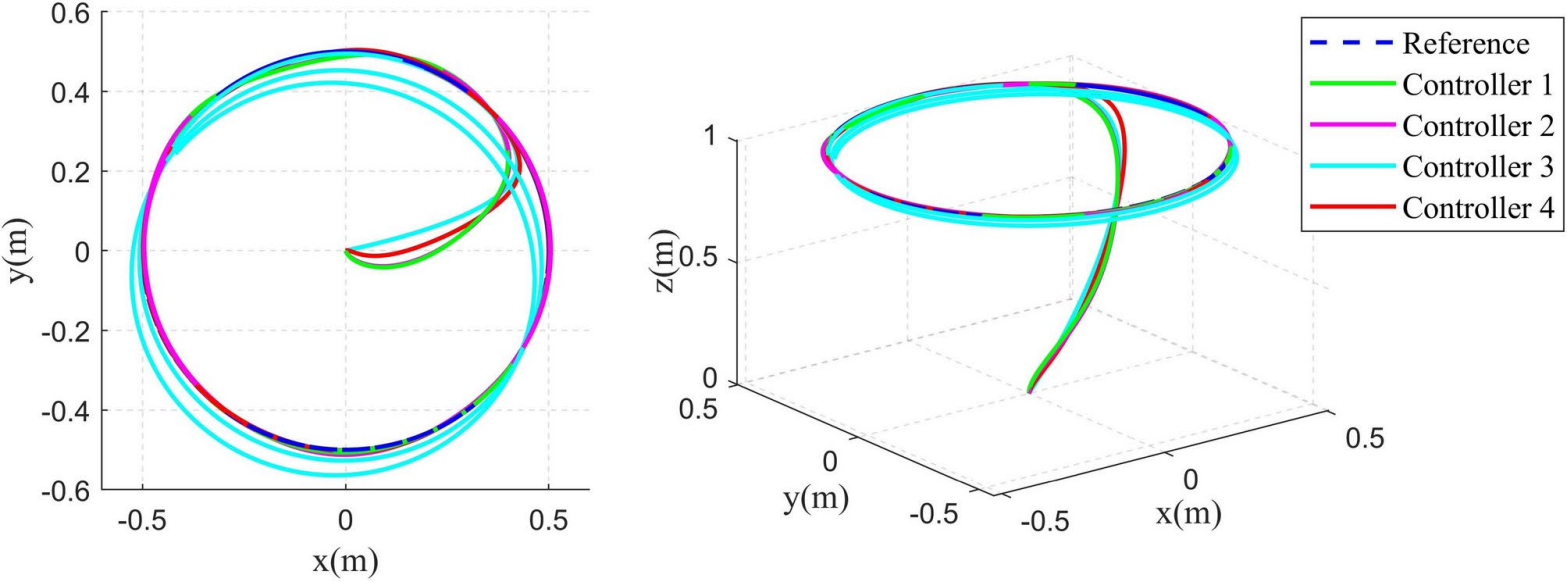
2D and 3D Diagrams of circular shape trajectory tracking.

**Fig. 7:**
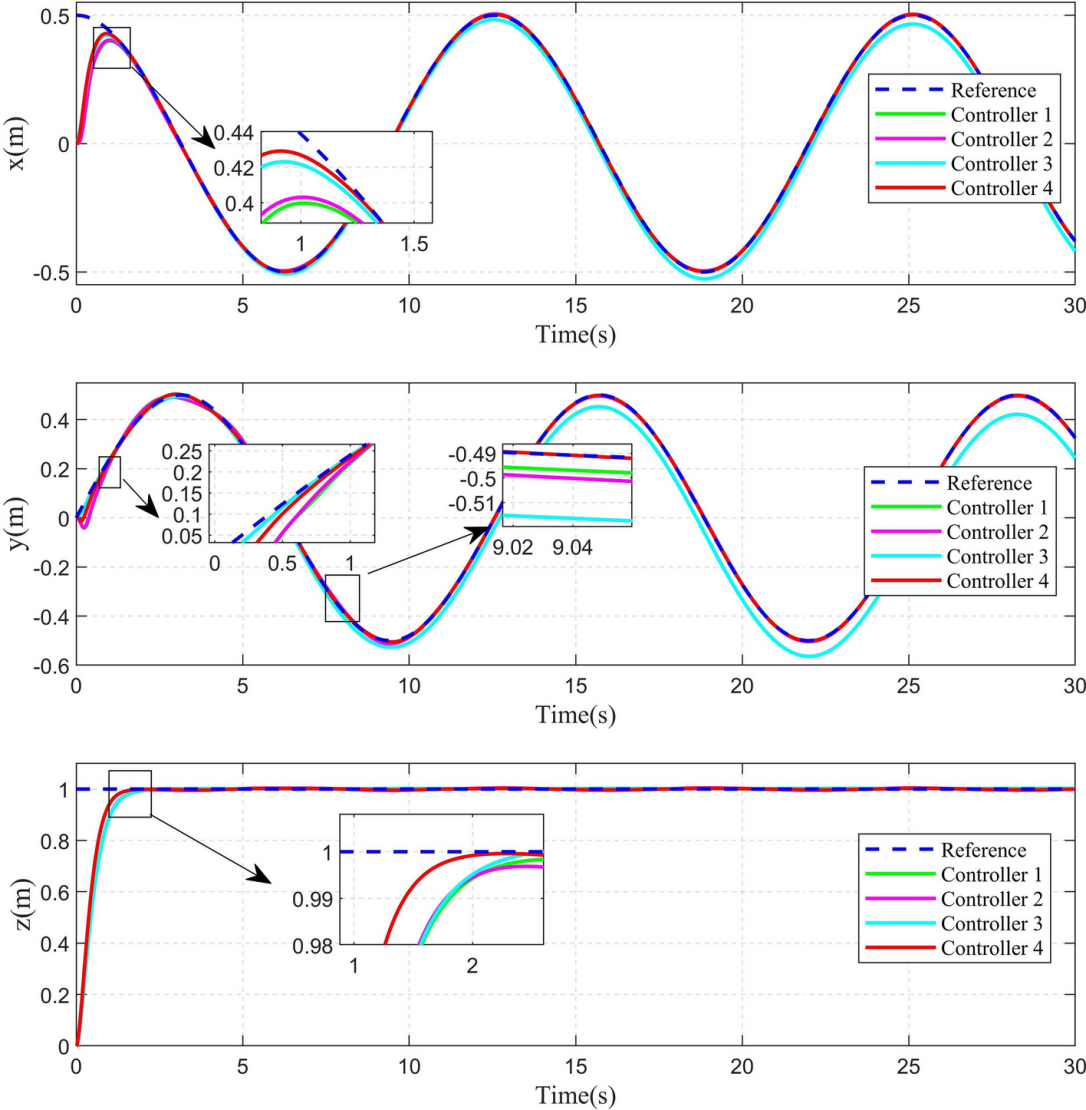
Position tracking curve of circular shape trajectory.

**Fig. 8:**
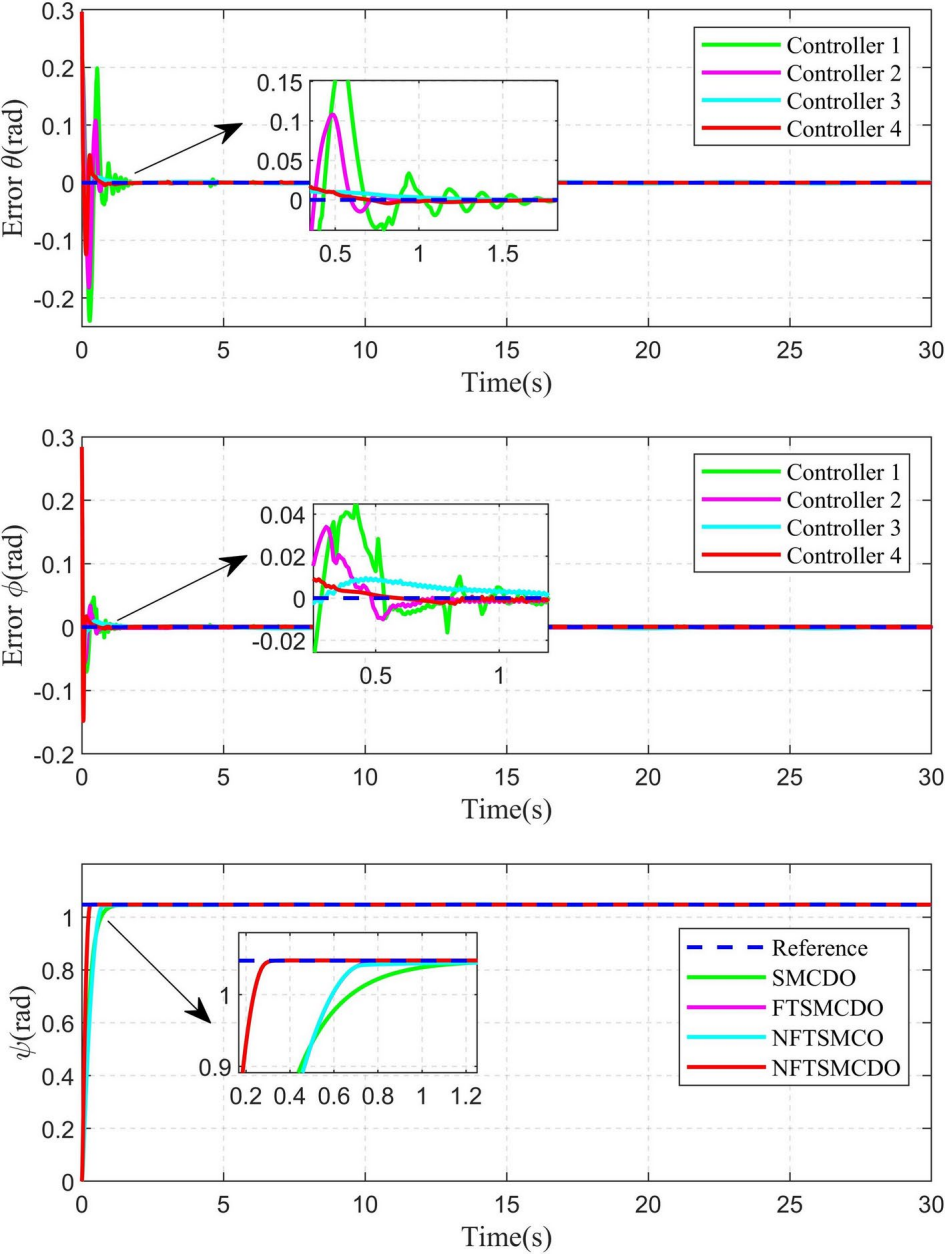
Attitude tracking error curve of circular shape trajectory.

**Fig. 9:**
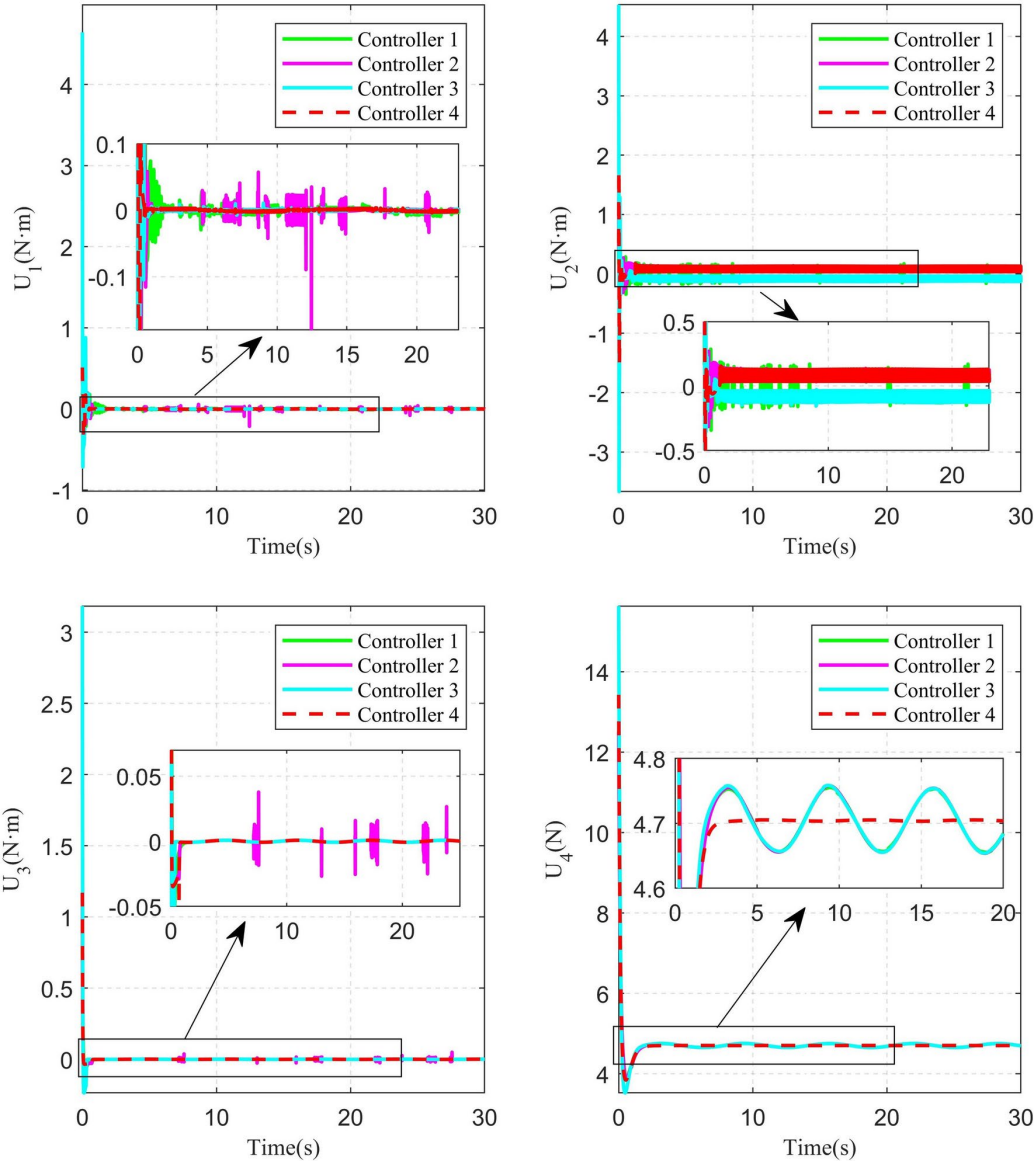
Control inputs of circular shape trajectory tracking.

Some comparisons of the controller proposed in this paper with some other controllers are given in the following. The first compared controller^33^, which combined the SMC with the DO, is referred to as Controller 1. The second compared controller^12^, which combined the FTSMC with the DO, is referred to as Controller 2. The third compared controller, which combined the NFTSMC with the time-varying delay observer, is referred to as Controller 3. The controller, which is proposed in this paper, is referred to as Controller 4. The simulation results of circular shape trajectory tracking are shown in Figs. 4, 5, 6, 7, 8 and 9. Figure 4 shows the performance of the disturbance observers in the attitude and position subsystems. It can be seen that the estimated disturbances in the attitude subsystem can converge fast to their real values. Although the estimated disturbances in the position subsystem exhibit some chatterings in the initial time, they can converge to their real values within 0.8s. The observation results of the time-varying delayed signals are shown in Fig. 5. It can be seen that the time-varying delay observer can successfully estimate the true values of the delayed signals.

The 2D and 3D trajectory tracking responses of the different controllers are shown in Fig. 6. It can be seen that, compared with the other three controllers, the controller proposed in this paper need less time to make the QUAV track the desired trajectory. The tracking performance of the position is shown in Fig. 7. From Fig. 7, it can be seen that, compared with the other three controllers, the controller 4, proposed in this paper, can achieve the fastest response speed. Moreover, the controller 3 without the disturbance observers has significant tracking errors in both position x and position y (the maximum errors is 0.04m and 0.08m in position x and position y, respectively). Fig. 8 shows the tracking errors of $\theta$, $\phi$, $\psi$. It can be seen that, compared to the other three controllers, the controller 4, proposed in this paper can achieve the fastest attitude tracking, and least system chattering. In order to make a quantitative comparison of these control strategies, the integral absolute error (IAE) and integral squared error (ISE) performance metrics for position and attitude tracking are given in Tables 3 and 4, respectively. From the tables, it can be seen that, compared to the other controllers, the controller proposed in this paper has the minimum IAE and ISE. The control inputs of the four controllers are illustrated in Figure 9. It can be seen that, compared with the other three controllers, the controller, proposed in this paper, can reach the minimum value of control signal chattering.

**Table 3:** Comparison of index performance (IAE) of circular shape trajectory tracking.

State	Controller 1	Controller 2	Controller 3	Controller 4
*x*	0.3018	0.3228	0.7972	0.2280
*y*	0.1576	0.1795	1.3008	0.0827
*z*	0.5673	0.6014	0.5721	0.4904
$\theta$	0.0999	0.0643	0.0661	0.0331
$\phi$	0.0395	0.0302	0.0459	0.0243
$\psi$	0.2620	0.2690	0.2964	0.1154

**Table 4:** Comparison of index performance (ISE) of circular hape trajectory racking.

State	Controller 1	Controller 2	Controller 3	Controller 4
*x*	0.0823	0.0799	0.0785	0.0617
*y*	0.0052	0.0049	0.0751	0.0011
*z*	0.3238	0.3245	0.3267	0.2783
$\theta$	0.0145	0.0077	0.0061	0.0029
$\phi$	0.0018	0.0013	0.0029	0.0017
$\psi$	0.1591	0.1736	0.1737	0.0771

### Example 2

To further validate the effectiveness and advantages of the proposed control algorithm, an infinity shape reference trajectory is introduced, and the parameters of the QUAV are the same as those in example 1. The infinity shape reference trajectory and the yaw angle are designed as following:

$$\begin{aligned} \left\{ \begin{aligned} {x_d}&= \frac{1}{2}\sin (\frac{t}{4})\\ {y_d}&= \frac{1}{2}\sin (\frac{t}{2})\\ {z_d}&= 3 - \sin (\frac{t}{4})\\ {\psi _d}&= \frac{\pi }{3} \end{aligned} \right. \end{aligned}$$

**Fig. 10:**
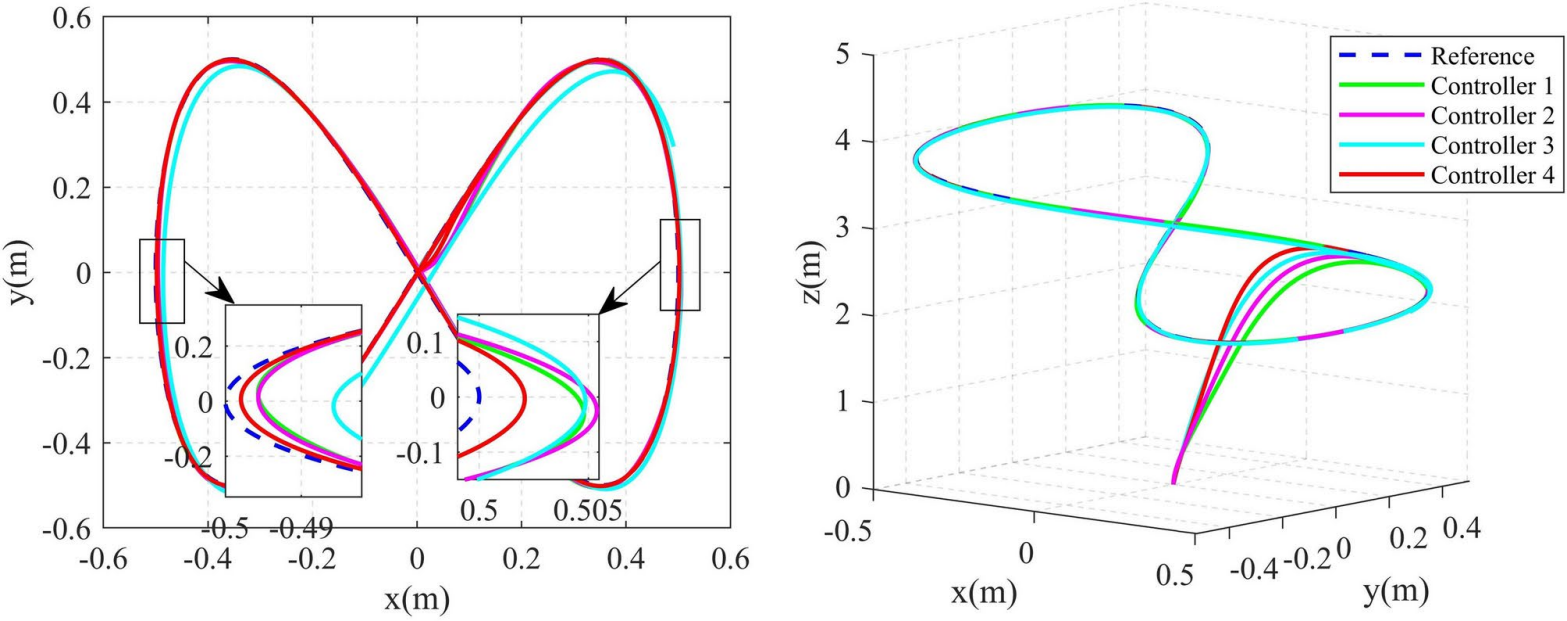
2D and 3D Diagrams of infinity shape Trajectory Tracking.

**Fig. 11:**
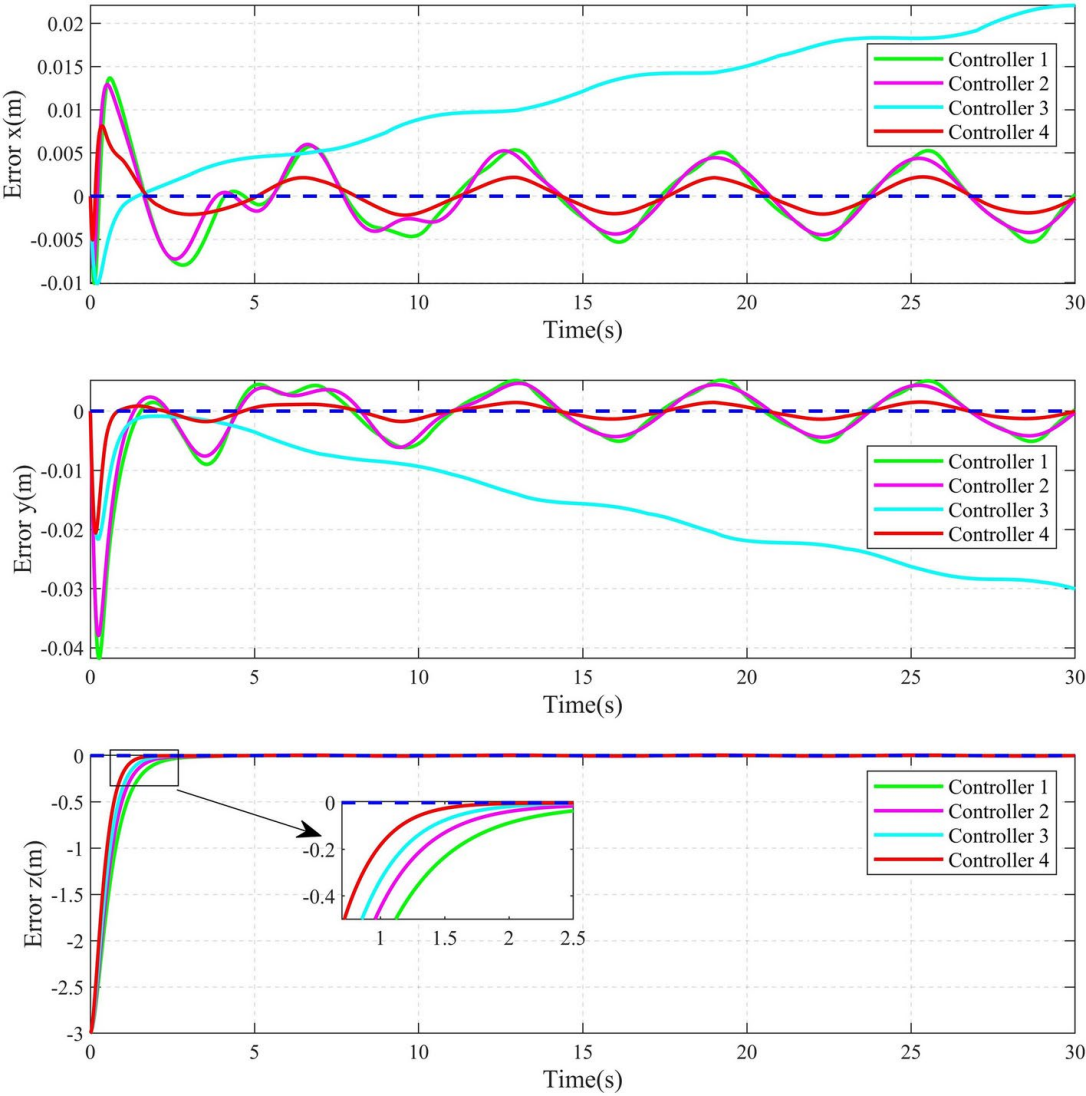
Position tracking error curve of infinity shape trajectory.

**Fig. 12:**
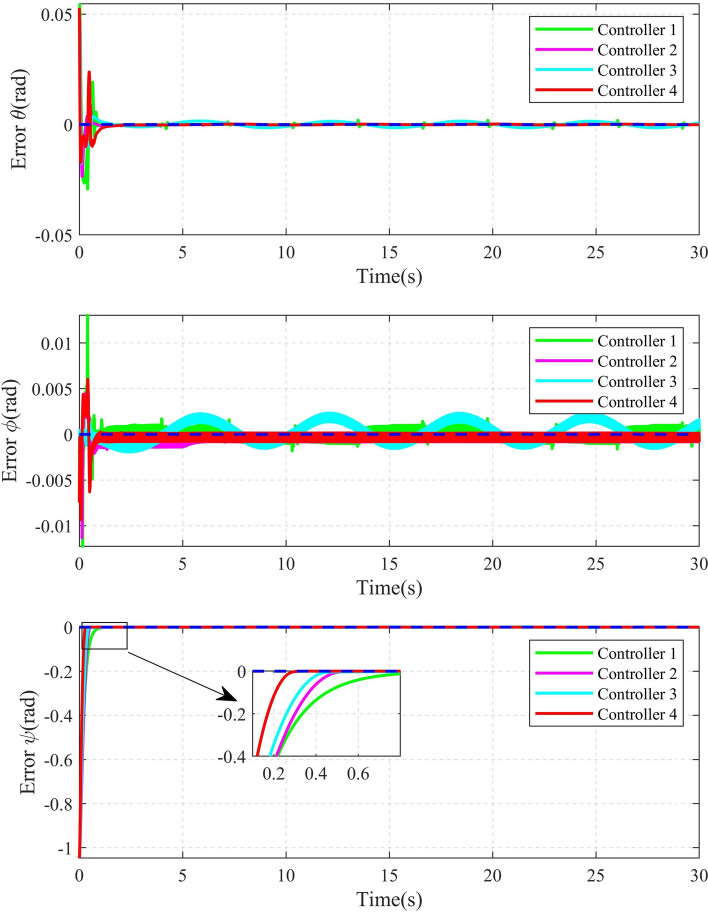
Attitude tracking error curve of infinity shape trajectory.

After doing the simulation by computer. The 2D and 3D trajectory tracking results are shown in Fig. 10. It can be seen that the controller 4 obtained in this paper can achieve the fastest response speed, compared with the other three controllers. Figure 11 shows the position tracking error curves. Compared to other three methods, the error x and error y curves of the proposed method are smoother, less volatile, and closer to zero. Taking the error x curve as an example, the maximum errors of controllers 1-4 are about 0.0138m, 0.0129m, 0.0221m and 0.0081m,respectively. The maximum error of Controller 4 is about 41.30%, 37.21%, and 63.35% lower than that of Controller 1, Controller 2, and Controller 3, respectively. Figure 12 shows the attitude tracking error curves. It can be seen that, compared with the other three controllers, the controller 4 proposed in this paper exhibits smaller error fluctuations. A more detailed comparisons of position and attitude tracking errors are shown in Tables 5 and 6. From the two tables, it can be seen that the controller 4 proposed in this paper has the minimum IAE and ISE, that means, the controller 4 achieves the highest tracking accuracy. These former results can validate the effectiveness and superiority of the controller 4 proposed in this paper, obviously.

**Table 5:** Comparison of index performance (IAE) of infinity shape trajectory tracking.

State	Controller 1	Controller 2	Controller 3	Controller 4
*x*	0.0989	0.0927	0.3525	0.0437
*y*	0.1147	0.1049	0.4691	0.0318
*z*	2.1367	1.8490	1.5921	1.3734
$\theta$	0.0162	0.0139	0.0338	0.0131
$\phi$	0.0152	0.0151	0.0284	0.0125
$\psi$	0.2271	0.2003	0.1939	0.1155

**Table 6:** Comparison of index performance (ISE) of infinity shape trajectory tracking.

State	Controller 1	Controller 2	Controller 3	Controller 4
*x*	0.000487	0.000414	0.005286	0.000096
*y*	0.001080	0.000874	0.009751	0.000125
*z*	3.684500	3.219300	2.904900	2.478300
$\theta$	0.000350	0.000141	0.000138	0.000121
$\phi$	0.000018	0.000016	0.000036	0.000015
$\psi$	0.139930	0.134790	0.112060	0.077074

## Conclusion

In this paper, a DO-based NFTSMC strategy is presented to solve the trajectory tracking problem for the QUAVs under unknown disturbances and time-varying delays. Firstly, considering the response of the attitude is faster than that of the position for a QUAV, the QUAVs’ model is decoupled into two subsystems: the position subsystem and the attitude subsystem, and then a hierarchical control strategy, which includes the inner and outer loops, is given to control the two subsystems. Secondly, considering that the position data are typically obtained through the GPS module, which introduces significant time delays during signal transmission and processing. Thus, the time-varying delays are considered in the position subsystem, and uncertain disturbances are considered in both subsystems. Thirdly, for the position subsystem (outer loop), a sliding mode controller is presented to control the position of the QUAVs. For the attitude subsystem (inner loop), a novel NFTSMC,which includes an exponential term, is introduced to ensure the fast convergence of the attitude angles. Based on the exponential term, the singularity problem of the conventional terminal sliding mode is solved. In the end, the effectiveness and superiority of the proposed control scheme are demonstrated by some numerical simulations. Specifically, the maximum position tracking error of the proposed controller in this paper is approximately 0.0081m, which is 41.30% and 37.21% lower than that of SMC and TSMC, respectively. However, there are still some further investigations, such as the fault-tolerant control, Network-based control, etc., that are not contained in this paper, and they will be the authors’future work.

## Data Availability

The datasets generated and/or analysed during the current study are not publicly available due to the confidentiality requirements of our laboratory but are available from the corresponding author on reasonable request.
